# Ectopic endometriosis, menstruation, and acute appendicitis: A thought-provoking case

**DOI:** 10.1016/j.ijscr.2021.01.099

**Published:** 2021-02-01

**Authors:** Tomohide Hori, Hideki Harada, Michihiro Yamamoto, Masahiro Yamada, Takefumi Yazawa, Ben Sasaki, Masaki Tani, Hikotaro Katsura, Asahi Sato, Yudai Sasaki, Hidekazu Yamamoto

**Affiliations:** Department of Surgery, Shiga General Hospital, Moriyama, 5-4-30 Moriyama, Moriyama, Shiga 524-8524, Japan

**Keywords:** Endometriosis, Endometrium, Ectopic endometrial gland, Menstruation, Appendix, Appendicitis

## Abstract

**Introduction and importance:**

Ectopic endometrium in the appendix is rare. The relationships between ectopic endometrium in the alimentary tract and digestive symptoms and between digestive symptoms due to ectopic endometriosis and periodic menstruation are controversial. We herein describe the successful treatment of acute appendicitis that we suspect was caused by ectopic endometriosis and periodic menstruation.

**Presentation of case:**

A 38.9-year-old multipara with uterine didelphys developed lower abdominal pain during menstruation, and she was clinically diagnosed with acute appendicitis. She received conservative management with cephem antibiotics, and her pain disappeared uneventfully. However, the lower abdominal pain during menstruation later recurred, and she again received conservative treatment. Laparoscopic appendectomy was subsequently performed because for 4 months, her appendicitis-induced digestive symptoms had recurred in association with periodic menstruation. Ectopic endometrial gland proliferations were histopathologically observed in the proper muscular layer of the appendiceal tip. She developed no further episodes of digestive symptoms postoperatively.

**Clinical discussion:**

Ectopic endometriosis of the alimentary tract may be accompanied by digestive symptoms; moreover, these symptoms may be related to periodic menstruation. However, the sensitivity of ectopic endometrium to hormones shows considerable variation among patients. We speculate that the acute appendicitis might have been triggered by ectopic endometriosis in our case because the patient developed repeated digestive symptoms in association with periodic menstruation. Ectopic endometrium may be incidentally observed in histopathological assessments of resected specimens. The therapeutic strategy should be carefully decided on a case-by-case basis.

**Conclusion:**

We hope this thought-provoking case provides a timely reminder for gastrointestinal clinicians and general surgeons.

## Highlights

• Ectopic endometrium in the appendix is rare at only 0.005 %.
• The association between digestive symptom and ectopic endometrium is controversial.
• The relationship between digestive symptoms and periodic menstruation is unclear.
• The sensitivity to hormones has considerable variation among patients.
• Ectopic endometriosis and periodic menstruation may trigger acute appendicitis.

## 1 Introduction

Whether ectopic endometrium of the alimentary tract may be associated with digestive symptoms (*e.g.*, pain, vomiting, and melena) remains controversial [1,2], and whether digestive symptoms in these patients are related to periodic menstruation is unclear [[3], [4], [5]]. Ectopic endometrium in the appendix was first documented by Sampson [6] in 1922, and its incidence is rare at only 0.005 % [1]. Ectopic endometriosis and periodic menstruation may trigger acute appendicitis [3], which generally requires prompt surgery [7].

We herein describe the successful treatment of a patient with acute appendicitis that we suspected was caused by ectopic endometriosis and periodic menstruation. This case was reported in accordance with the SCARE 2020 Guideline [8].

## 2 Presentation of case

**Fig. 1:**
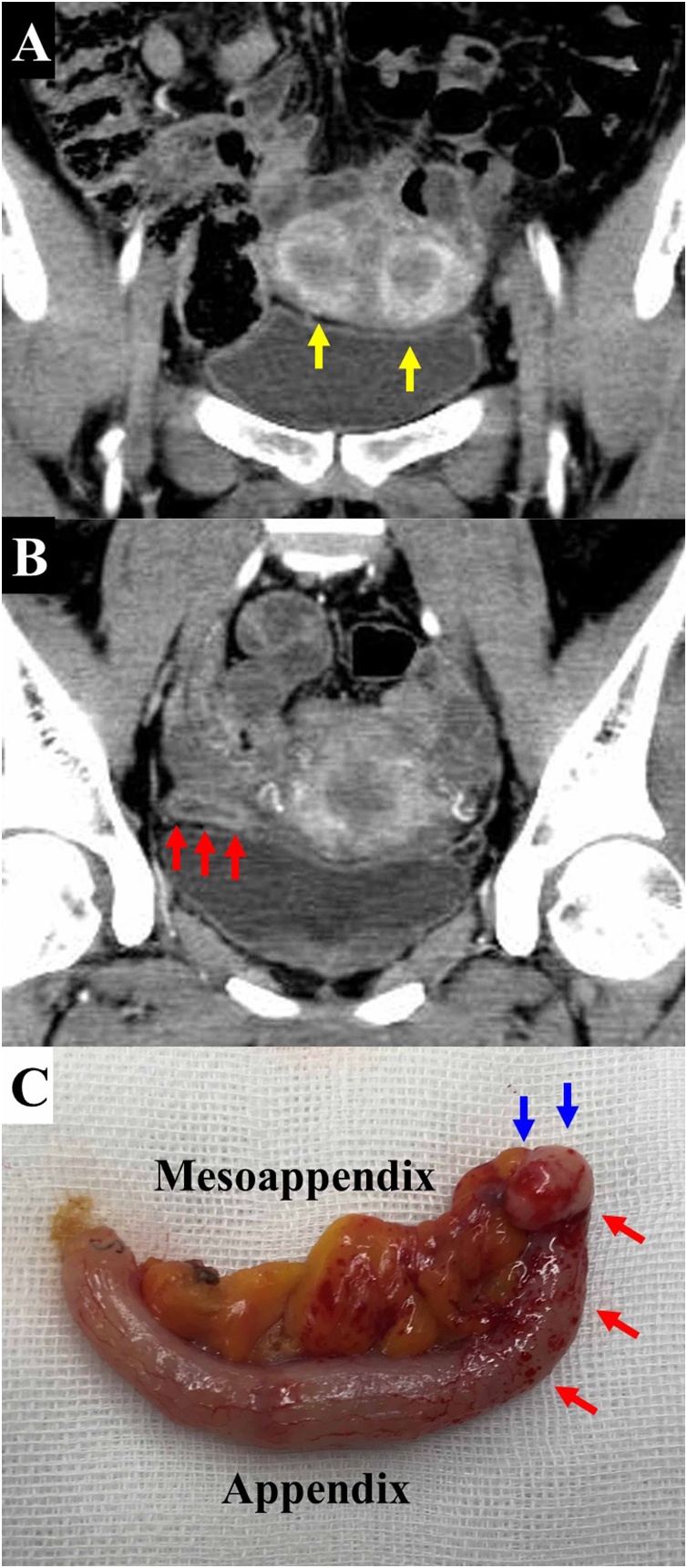
**Findings of image study and resected specimen.** (A) The patient, a multiparous woman, had uterine didelphys **(yellow arrows)**. (B) A swollen appendix with thickened walls was clearly detected with contrast enhancement **(red arrows)**. (C) Inflammatory change was observed in the appendiceal body **(red arrows)**, and an elastic hard induration was palpated in the appendiceal tip **(blue arrows)**.

**Fig. 2:**
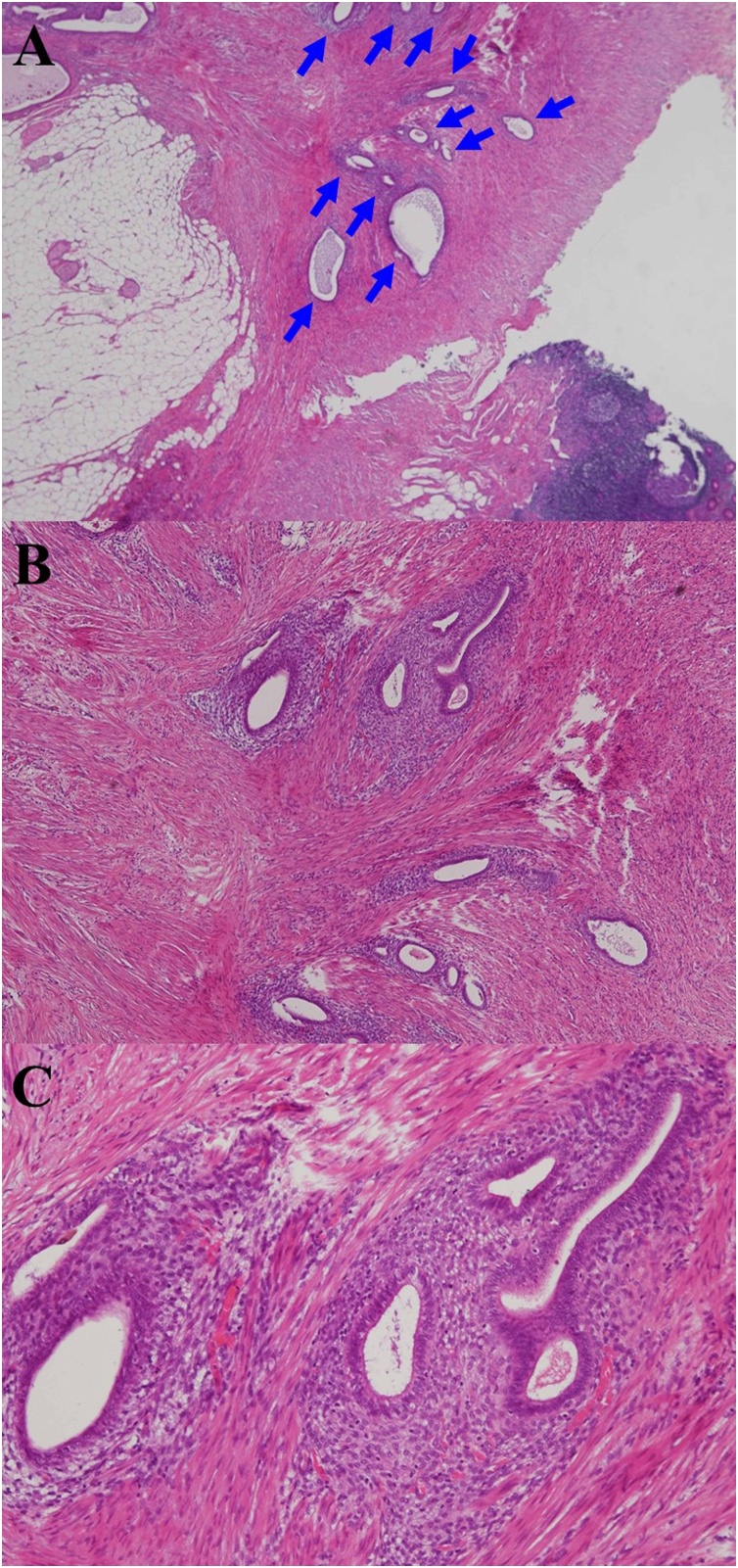
**Histopathological findings.** Histopathological findings obtained by hematoxylin and eosin staining are shown at magnifications of (A) ×20, (B) ×100, and (C) ×200. (A) Endometrial gland proliferations were ectopically observed in the proper muscular layer of the appendiceal tip **(blue arrows)**.

The patient was a 38.9-year-old multipara with uterine didelphys (Fig. 1A) and no known ovarian diseases. She developed lower abdominal pain during menstruation, and a clinical diagnosis of acute appendicitis was made based on blood examination results and contrast-enhanced computed tomography findings (Fig. 1B). She received conservative management with cephem antibiotics, and her pain disappeared uneventfully. However, the pain later recurred during menstruation, and she again received conservative treatment. Laparoscopic appendectomy with a minimized port incision [7] was subsequently performed because for 4 months, her appendicitis-induced digestive symptoms recurred in association with periodic menstruation. An elastic hard induration was palpated in the appendiceal tip of the resected specimen (Fig. 1C). The patient’s postoperative course was uneventful, and she was discharged from the hospital on postoperative day 2. Ectopic endometrial gland proliferations were histopathologically observed in the muscularis propria (proper muscular layer) of the appendiceal tip (Fig. 2). The patient developed no further episodes of digestive symptoms after the elective surgery.

## 3 Discussion

Ectopic endometriosis of the alimentary tract may be accompanied by digestive symptoms [1]. Intestinal invagination and perforative peritonitis have been observed in these patients [1]. However, this ectopic endometrium may have no relationship with digestive symptoms [2]. Although one-third of patients with ectopic endometrium in the appendix have submucosal lesions [9], there might be no relationship between the location of the ectopic endometrium in the appendix and digestive symptoms due to ectopic endometriosis [9]. Thus, the association between digestive symptom and ectopic endometrium remains unclear. We speculate that the acute appendicitis might have been triggered by ectopic endometriosis in our case because the patient developed recurrent digestive symptoms in association with periodic menstruation.

Digestive symptoms due to ectopic endometriosis in the appendix may have a relationship with periodical menstruation [3], or they may not [4,5]. In contrast to ectopic endometriosis in the ovary, ectopic endometriosis in the appendix may cause digestive symptoms even after menopause [10]. Prompt surgery is generally required for acute appendicitis [7], though our patient underwent elective surgery. A possible explanation for this inconsistency is that the sensitivity of ectopic endometrium to hormones has considerable variation among patients [4,5]. Ectopic endometriosis and periodic menstruation may trigger acute appendicitis [3], and our case supports this hypothesis.

The pathogenesis of ectopic endometrium in the appendix is still unknown, although ectopic transplantation *via* the oviduct has been suggested [2]. Ectopic proliferations of endometrial glands were histopathologically observed in the proper muscular layer of the appendiceal tip. The tip is the most frequent site of such proliferation [1], supporting the hypothesis of pathogenesis of ectopic transplantation *via* the oviduct [6]. Notably, however, some researchers have reported that the body of the appendix is the most frequent site [11]. In contrast, ectopic proliferations of endometrial glands are generally observed in the muscularis propria or subserosal layer [1], supporting the hypothesis of pathogenesis of ectopic transplantation [1]. Most patients with ectopic endometrium in the appendix actually have no ovarian diseases [9]. Consistent with this, no ovarian diseases were observed in our patient. This finding may oppose the hypothesis of pathogenesis of ectopic transplantation *via* the oviduct, and some researchers have suggested other possible explanations (*e.g.*, epithelial metaplasia and metastatic transplantation) [12].

In general, the therapeutic strategy for ectopic endometriosis is decided based on the patient’s hope for timing of pregnancy and childbirth and the disease severity [13]. Supportive care, medical therapy, and surgical treatment are options, and the main medical treatment is hormonal therapy [13]. Ectopic endometrium has been linked with carcinogenesis [10]. Ectopic endometrium may also be incidentally observed in histopathological assessments of resected specimens. Treatment strategies should be decided on a case-by-case basis. The primary physician and a gynecologist should collaboratively deliberate additional surveys for ectopic endometrium in other organs and the optimal therapeutic strategy in the postoperative period.

## 4 Conclusions

We have herein presented a successfully treated case of acute appendicitis suspected to have been caused by ectopic endometriosis and periodic menstruation. We hope our thought-provoking case will provide a timely reminder for gastrointestinal clinicians and general surgeons.

## Declaration of Competing Interest

None of the authors have any financial conflicts of interest to declare.

## Funding

The authors declare that they received no funding support for this report.

## Ethical approval

Data were retrospectively evaluated. This report was approved by the Institutional Review Board of Shiga General Hospital, Moriyama, Japan.

## Consent

Written informed consent was obtained from the patient for publication of this case report and accompanying images. A copy of the written consent is available for review by the Editor-in-Chief of this journal on request.

## Author contribution

Tomohide Hori, PhD., MD., FACS. clinically managed treatments including preoperative diagnosis, and actually performed laparoscopic surgery with minimized port incision. T. Hori analyzed the data, and wrote the manuscript. All authors discussed therapeutic options, reviewed previous papers, and provided important opinions. T. Hori supervised this report.

## Registration of research studies

Not applicable.

## Guarantor

None.

## Provenance and peer review

Not commissioned, externally peer-reviewed.
